# Supplementary material for: Reconciling Mining with the Conservation of Cave Biodiversity: A Quantitative Baseline to Help Establish Conservation Priorities
Source: PLoS One. 2016 Dec 20;11(12):e0168348. doi: 10.1371/journal.pone.0168348 (PMC5173368; doi:10.1371/journal.pone.0168348)
Supplement: S1 Dataset — (ZIP) [file pone.0168348.s002.zip › Taxa/Serra Norte/SN_2013/N4WS/Lista N4WS-55.pdf]

CAVIDADE N4WS-0055

Classe	Ordem	Fam/Outros	Gên/Outros	Espécie	S-U	U-S	Zon.
Arachnida	Acari	(Parasitengonina)		sp.1		X	E
Arachnida	Acari	(Parasitengonina)		sp.5	X		P
Arachnida	Acari	(Uropodina)		sp.4	X		P
Arachnida	Acari	Argasidae	*Ornithodoros*	sp.1		X	E
Arachnida	Acari	Labidostommatidae		sp.1	X	X	E/P
Arachnida	Acari	Mesostigmata		jovem		X	E
Arachnida	Acari	Prostigmata		sp.3	X		P
Arachnida	Acari	Rhagidiidae		sp.1		X	E
Arachnida	Acari	Tydeidae		sp.1	X		P
Arachnida	Acari	Veigaiidae		sp.1	X		P
Arachnida	Amblypygi	Phrynidae	*Heterophrynus*	*longicornis*	1		E
Arachnida	Araneae	Actinopididae	*Actinopus*	sp.1		1	E
Arachnida	Araneae	Araneidae		jovem	X		P
Arachnida	Araneae	Corinnidae		sp.2	1	1	E
Arachnida	Araneae	Ctenidae	*Enoploctenus*	sp.1	1		E
Arachnida	Araneae	Ctenidae		jovem	1	1	E/P
Arachnida	Araneae	Mimetidae		jovem		X	E
Arachnida	Araneae	Mysmenidae	*Microdipoena*	sp.1	X		E
Arachnida	Araneae	Nesticidae		sp.1	X		P
Arachnida	Araneae	Ochyroceratidae	*Speocera*	sp.1	X	X	E/P
Arachnida	Araneae	Oonopidae		jovem	X		P
Arachnida	Araneae	Pholcidae	*Mesabolivar*	*aurantiacus*	X		P
Arachnida	Araneae	Pholcidae	*Mesabolivar*	*eberhard*	X		P
Arachnida	Araneae	Pholcidae	Ninetinae	sp.1	X	X	E/P
Arachnida	Araneae	Salticidae		jovem	X		P
Arachnida	Araneae	Scytodidae	*Scytodes*	jovem	1	1	E/P
Arachnida	Araneae	Symphytognathidae	*Anapistula*	sp.1		X	E
Arachnida	Araneae	Theraphosidae		jovem	3	3	E/P
Arachnida	Araneae	Theridiidae		jovem	X	X	E/P
Arachnida	Araneae	Theridiidae		sp.2		X	E
Arachnida	Araneae	Theridiidae		sp.3		X	E
Arachnida	Araneae	Theridiidae		sp.5		X	E
Arachnida	Araneae	Theridiosomatidae	*Plato*	sp.1	X	X	E/P
Arachnida	Opiliones	Escadabiidae		sp.3		X	E
Arachnida	Opiliones	Sclerosomatidae	*Prionostema*	sp.1		X	E
Arachnida	Opiliones	Stygnidae		jovem		1	E
Arachnida	Palpigradi	Eukoeneniidae	*Leptokoenenia*	*thalassophobica*	X		P
Arachnida	Pseudoscorpiones	Chernetidae	*Spelaeochernes*	sp.1	X	X	E/P
Arachnida	Pseudoscorpiones	Chthoniidae	*Pseudochthonius*	sp.1	X	X	E/P
Arachnida	Pseudoscorpiones	Olpiidae		jovem		X	E
Arachnida	Schizomida	Hubbardiidae	aff. *Rowlandius*	sp.1		X	E
Chilopoda	Scolopendromorpha	Scolopocryptopidae	*Newportia*	sp.1		1	E
Chilopoda	Scutigeromorpha	Pselliodidae		jovem		4	E
Diplopoda	Polydesmida	Tridontomidae		sp.1		X	E
Diplopoda	Polyxenida	Hypogexenidae		sp.1		X	E
Diplopoda	Spirostreptida	Pseudonannolenidae	*Pseudonannolene*	sp.1	1	1	E
Entognatha	Collembola	Entomobryidae		sp.6		X	E
Entognatha	Collembola	Isotomidae		sp.1		X	E
Entognatha	Collembola	Paronellidae		sp.1	X	X	E

Entognatha	Collembola	Symphypleona		sp.1	X	X	E
Entognatha	Diplura	Campodeidae		sp.1	X	X	E
Entognatha	Diplura	Japygidae		sp.1		X	E
Entognatha	Diplura	Projapygidae		sp.1	X	X	E
Entognatha	Diplura	Projapygidae		sp.2		X	E
Insecta	Blattodea			jovem	7	1	E
Insecta	Coleoptera	Carabidae		jovem		X	E
Insecta	Coleoptera	Elateridae		jovem		X	E
Insecta	Coleoptera	Scarabaeidae		jovem		X	E
Insecta	Coleoptera			jovem	X	X	E
Insecta	Dermaptera	Anisolabididae		jovem	X		
Insecta	Diptera	Calliphoridae	Crysomyinae	sp.	X		
Insecta	Diptera	Cecidomyiidae	Cecidomyiinae	sp.	X		
Insecta	Diptera	Ceratopogonidae		sp.	X		
Insecta	Diptera	Chironomidae		sp.		X	E
Insecta	Diptera	Ephydridae		sp.	X		
Insecta	Diptera	Phoridae	Metopininae	sp.		X	E
Insecta	Diptera	Psychodidae	*Sciopemyia*	*sordellii*	X	X	E
Insecta	Diptera	Sciaridae	*Bradysia*	sp.	X		
Insecta	Diptera	Sciaridae	*Epidapus*	sp.		X	E
Insecta	Diptera	Streblidae	*Strebla*	sp.		X	E
Insecta	Diptera	Tipulidae	Tipulinae	sp.	X		
Insecta	Heteroptera	Reduviidae	Reduviinae	jovem	54	1	E
Insecta	Heteroptera	Schizopteridae	Schizopterinae	jovem	X		
Insecta	Homoptera	Cicadellidae		sp.4	X		
Insecta	Homoptera	Cixiidae		jovem	X	X	E
Insecta	Hymenoptera	Braconidae		sp.1		X	E
Insecta	Hymenoptera	Formicidae	*Anochetus*	sp.1		X	E
Insecta	Hymenoptera	Formicidae	*Camponotus*	sp.1		X	E
Insecta	Hymenoptera	Formicidae	*Gnamptogenys*	*striatula*		X	E
Insecta	Hymenoptera	Formicidae	*Nylanderia*	sp.2		X	E
Insecta	Hymenoptera	Formicidae	*Octostruma*	sp.1	X		
Insecta	Hymenoptera	Formicidae	*Pachycondyla*	*striata*	X		
Insecta	Hymenoptera	Formicidae	*Pheidole*	sp.2	X		
Insecta	Hymenoptera	Formicidae	*Solenopsis*	sp.1	X		
Insecta	Hymenoptera	Formicidae	*Solenopsis*	sp.2		X	E
Insecta	Isoptera	Termitidae	*Cortaritermes*	*silvestrii*	X		
Insecta	Isoptera	Termitidae	*Embiratermes*	sp.	X	X	E
Insecta	Isoptera	Termitidae	*Nasutitermes*	sp.	X		
Insecta	Isoptera	Termitidae	*Spinitermes*	sp.	X		
Insecta	Lepidoptera	Noctuoidea		sp.1	1		E
Insecta	Lepidoptera			jovem	X	X	E
Insecta	Neuroptera	Myrmeleontidae		jovem	4	1	E
Insecta	Orthoptera	Phalangopsidae	*Paraclodes*	sp.	5		E
Insecta	Orthoptera	Phalangopsidae	*Phalangopsis*	sp.	49	6	E/P
Insecta	Psocoptera	Epipsocidae	*Epipsocus*	sp.1		X	E
Insecta	Psocoptera	Manicapsocidae	*Nothoentomum*	sp.1		X	E
Insecta	Psocoptera	Psyllipsocidae		jovem		X	E
Gastropoda	Pulmonata	Systrophiidae	*Happia*	sp.		X	E
Malacostraca	Isopoda	Armadillidae		sp.1		X	E
Malacostraca	Isopoda	Philosciidae		sp.1	X	X	E
Malacostraca	Isopoda	Philosciidae		sp.2	X	X	E

Malacostraca	Isopoda	Scleropactidae	*Circoniscus*	*buckupi*	X		
Pauropoda	Tetramerocerata			sp.1		X	E
Amphibia	Anura	Dendrobatidae	*Epipedobates*	sp.	1		E
Amphibia	Anura	Strabomantidae	*Pristimantis*	cf. *fenestratus*	12	1	E
Mammalia	Chiroptera	Emballonuridae	*Peropteryx*	sp.	13	30	E
Mammalia	Chiroptera	Furipteridae	*Furipterus*	*horrens*		10	
Mammalia	Chiroptera	Phyllostomidae	*Carollia*	*perspicillata*	2	6	E
Mammalia	Rodentia	Cricetidae	*Rhipidomys*	sp.	2		E
